# A Cyanophage MarR-Type Transcription Factor Regulates Host RNase E Expression during Infection

**DOI:** 10.3390/microorganisms10112245

**Published:** 2022-11-13

**Authors:** S. Joke Lambrecht, Nils Stappert, Frederik Sommer, Michael Schroda, Claudia Steglich

**Affiliations:** 1 Faculty of Biology, University of Freiburg, 79104 Freiburg, Germany; 2 Medical Faculty, Medical Center, Institute for Surgical Pathology, University of Freiburg, 79106 Freiburg, Germany; 3 Molecular Biotechnology & Systems Biology, TU Kaiserslautern, 67663 Kaiserslautern, Germany

**Keywords:** MarR family transcription factor, cyanophage, *Prochlorococcus*, transcriptional regulation of RNase E

## Abstract

The marine picocyanobacterium *Prochlorococcus* contributes significantly to global primary production, and its abundance and diversity is shaped in part by viral infection. Here, we identified a cyanophage-encoded MarR-type transcription factor that induces the gene expression of host *Prochlorococcus* MED4 endoribonuclease (RNase) E during phage infection. The increase in *rne* transcript levels relies on the phage (p)MarR-mediated activation of an alternative promoter that gives rise to a truncated yet enzymatically fully functional RNase E isoform. In this study, we demonstrate that pMarR binds to an atypical activator site downstream of the transcriptional start site and that binding is enhanced in the presence of Ca^2+^ ions. Furthermore, we show that dimeric pMarR interacts with the α subunit of RNA polymerase, and we identified amino acid residues S66, R67, and G106, which are important for Ca^2+^ binding, DNA binding, and dimerization of pMarR, respectively.

## 1. Introduction

The most abundant photosynthetic organism on Earth is the marine picocyanobacterium *Prochlorococcus*, which thrives in the vast ocean regions between 40° N and 40° S, reaching an estimated global population size of 3 × 10^27^ cells [1,2] and therefore contributes substantially to the marine primary production and biogeochemical cycles [3]. The population size and diversity, the transfer of genetic material between cells, and the recycling of nutrients and organic carbon in the aquatic system is tremendously influenced by coexisting marine viruses, which every day kill approximately 20% of the living biomass [4]. Specifically for *Prochlorococcus,* a decline in abundance in the North Pacific Ocean has recently been attributed to viral infection [5].

The reproduction of phages heavily relies on their hosts, involving a multitude of interactions. Different types of phages have established different strategies for their efficient propagation. Phage proteins involved in the manipulation of host metabolism are often expressed in the early phase of infection and interact with host proteins or nucleic acids to inhibit, activate, or redirect the host target to serve their own purposes [6]. For example, the early gene product 0.7 of coliphage T7 (T7 Gp0.7) exhibits bifunctional activity, with the C-terminal part being involved in the shut-off of host transcription and the N-terminal to the middle region possessing a protein kinase activity [7]. This kinase activity results in the modification and, therefore, functional manipulation of over 90 host proteins [8]. One *E. coli* protein that is chemically modified by T7 Gp0.7, in concert with T7 Gp2 and T7 Gp5.7, is the RNA polymerase (RNAP) β subunit, leading to its complete shut off [9]. Another kinase target is endoribonuclease (RNase) E, whose cleavage activity on T7 mRNAs is attenuated by T7 Gp0.7 phosphorylation of the C-terminal half of RNase E [7]. This type of regulatory modification is not possible in cyanobacterial host-phage systems as the cyanobacterial RNase E/G family lacks the C-terminal half of the protein [10,11].

The marine cyanobacterium *Prochlorococcus* MED4 is prone to infection by the T7-like cyanopodovirus P-SSP7. In the first published genome annotation of P-SSP7 the early phage gene PSSP7_011 was annotated as *gp0.7* homologue based on genome comparison with T7 and its closest coliphage relatives T3, gh-1, ϕYe03–12, and ϕA1122 [12]. Although PSSP7_011 does not belong to the T7-like core genome, it is common within this group of phages. The protein ranges in size between 125 (P-SSP7) to 370 amino acids (T3 and ϕYe03-12) [12]. However, a previous genome comparison study of 20 marine cyanopodoviruses showed that homologues of PSSP7_011, although not part of the core genome, are nevertheless present in 7 of the 20 cyanophages [13]. Intriguingly, all of these homologues lack a protein kinase domain but possess a putative MarR transcriptional regulator domain. Notably, the closest homologue of PSSP7_011 is not found in another cyanophage but in the chromosomal DNA of the high light-adapted ecotype *Prochlorococcus* MIT0604. The *Prochlorococcus* MED4 genome also encodes a MarR homolog (PMM0363) located in genomic island 1, which shares 42% amino acid identity with PSSP7_011, but has been overlooked in previous work addressing the suite of transcriptional regulators in this organism [14].

Because of the missing kinase domain and the presence of a MarR domain in PSSP7_011 (from here on referred to as pMarR for phage MarR) it was suggested that this protein might be involved in redirecting transcription from the host towards the phage genome [15]. MarR proteins form homodimers and each subunit contains a winged helix-turn-helix DNA-binding domain, which mediates binding to palindromic repeats, typically leading to repression of transcription [16]. Homologs of MarR occur in more than 44,000 bacterial and archaeal genomes with an average copy number of seven per genome and modulate the gene expression of genes involved in multi-drug resistance, metabolism, stress responses, and virulence [16].

Previously, we observed an increase in the abundance of RNase E in *Prochlorococcus* MED4 in response to infection by cyanophage PSSP-7 [15,17]. In *E. coli* and many other bacteria, production of RNase E is tightly regulated by an autofeedback mechanism. The regulation is mediated in *cis* by the *rne* 5′ UTR, which senses the cellular level of RNase E and controls the decay rate of the *rne* message in response to changes in RNase E activity [18,19,20]. In *Prochlorococcus* MED4, an analogous autofeedback mechanism is by-passed during phage infection, as RNase E expression becomes driven by an alternative promoter giving rise to a transcript that lacks the native 5′ UTR and starts at G (+3) of the canonical ATG start codon, leading to a truncated, but enzymatically active RNase E isoform [17]. The bypass of RNase E autoregulation in combination with the almost complete coverage of phage transcripts with antisense RNAs that protect from RNase E cleavage, might shift ribolysis towards the host transcriptome during phage infection [17]. However, it remained unclear whether the switch in promoter usage is actively regulated by a phage factor or whether it is an indirect effect caused by the degradation of a host factor due to the shutdown of host gene expression during infection.

Here, we show that pMarR is a transcription factor positively regulating the alternative host *rne* promoter that drives the expression of the short RNase E variant. Binding of pMarR to the *rne* promoter was identified to occur at an unusual position downstream of the transcriptional start site (TSS), and pMarR-binding affinity increased almost 4-fold in the presence of calcium ions. Furthermore, we observed protein-protein interaction between pMarR and the alpha subunit of *Prochlorococcus* MED4 RNAP.

## 2. Materials and Methods

### 2.1. Growth Conditions of Prochlorococcus MED4

*Prochlorococcus* MED4 cultures were grown at 22 °C in AMP1 medium [21] under 30 µmol quanta m^−2^ s^−1^ of continuous white cool light to cell densities of 1–3 × 10^8^ cells per mL.

### 2.2. Generation of Expression Vectors

The promotor-GFP expression vectors were generated by PCR amplification of the pXG10-SF backbone including the coliphage T7 *gp10A* UTR [22] suitable for GFP expression but excluding the native promotor P_LtetO_ using primers # 77 and 78, and by PCR amplification of the DNA region of interest using primers # 80–87. The two fragments were joined by AQUA cloning [23]. Because MED4 promotors did not produce sufficient transcription for fluorescence measurements (Figure S1A), the original pXG10-SF promoter P_LtetO_ was reintroduced upstream of the DNA of interest using primers # 79, and 88–91.

The pQE70 expression vector for pMarR protein purification, protein pull-down, and GFP assays was generated using primers # 92–98 as a template for a codon optimized version of pMarR and the PCR amplified fragments were joined with the pQE70 backbone by AQUA cloning [23]. The mutant versions of pMarR were generated using primers # 1–32.

The backbones of the Y2H vectors pcGAD and pGBK were amplified using primers # 59–62 and genes of interest were amplified using primers # 39–58. The vector backbone and the inserts were joined by AQUA cloning [23], leading to a fusion protein with an AD or BD tag either at the N or C terminus.

### 2.3. E. coli GFP In Vivo Assay

Competent *E. coli* Top10 cells carrying the pREP4 plasmid of the M15 strain were cotransformed with the pXG10-SF plasmids encoding tested promoter regions in the upstream region of the GFP reporter gene and pQE70 plasmid for expression of pMarR or mutant versions. All cultivation steps of the GFP reporter assays were performed in the presence of chloramphenicol, ampicillin, and kanamycin. Transformed cells were streaked onto LB agar plates and incubated overnight. Colonies were transferred into 200 µL LB in a 96-well plate and incubated overnight at 37 °C with gentle agitation. A fresh 96-well plate was inoculated 1:100 with overnight grown cultures and incubated with gentle agitation for 2 h at 37 °C. The *pmarR* expression was induced by the addition of 1 mM IPTG and cells were further incubated for 3 h at 30 °C with gentle agitation. The cells were then fixed with 1% (final concentration) Histofix (Roth, Germany) and analyzed on the Accuri C6 flow cytometer (Beckman-Coulter, Brea, CA, USA) monitoring GFP fluorescence at an excitation wavelength of 488 nm and emission wavelength at 533/30 nm. Fold changes were calculated from the mean fluorescence of 50,000 cells per clone from 5–6 individual clones for the respective combinations.

### 2.4. Recombinant Protein Expression and Purification

pQE70 plasmids coding for pMarR or single amino acid mutant versions, all supplemented with a C-terminal his tag, were transformed into *E. coli* M15. Overnight grown cultures were diluted 1:100 into TB medium and grown for 2 h at 37 °C with agitation at 140 rpm. Recombinant protein expression was induced by the addition of 1 mM IPTG and cultures were further incubated for 3 h. The cells were collected by centrifugation at 4 °C for 10 min at 8000× *g*. The cell pellet was resuspended in lysis buffer (50 mM NaH_2_PO_4_, 1 M NaCl, 10% glycerol, 5 mM imidazole, 0.01% Tween20, cOmplete™ Protease Inhibitor Cocktail (Roche) and the cells were lysed using the One Shot constant cell disruption system (Constant Systems Limited, Daventry, UK) at 2.4 kbar in two consecutive cycles. The cellular debris was sedimented by centrifugation at 4 °C for 30 min at 14,000× *g*. The cell lysate was filtered through a 0.45 µm filter and loaded on a HisTrap HP column (Cytiva, Marlborough, MA, USA). The recombinant protein was purified and eluted by using a combination of washing buffer (5 mM Imidazole, 50 mM NaH_2_PO_4_, 300 mM NaCl, 5 mM NaN_3_) and elution buffer (800 mM Imidazole, 50 mM NaH_2_PO_4_, 300 mM NaCl). Fractions that contained the protein were pooled and the buffer was changed to TBS using a HiTrap desalting column (Cytiva, Marlborough, MA, USA) and concentrated using Amicon Ultra Centrifugal Filters 10K (Merck, Darmstadt, Germany). The protein concentration was determined using a Qubit (Thermo Scientific™, Waltham, MA, USA) and the final protein solution was substituted with 10% glycerol and 2 mM DTT for storage at 4 °C.

### 2.5. Electrophoretic Mobility Shift Assay (EMSA)

DNA sequences were amplified by PCR with fluorescein Cy5 labelled primers using Q5^®^ High-Fidelity Polymerase (NEB). Genomic DNA of *Prochlorococcus* MED4 and the cyanophage P-SSP7 were used as templates, respectively. The respective primers are listed in the Supplementary Table S1. All four *rne* segments P-, P, Pi, and Pi+ were Cy5 labeled. EMSAs were conducted using 12.5 ng of the target DNA, 1 µg of Lightshift™ Poly dI-dC (Thermo Scientific™, Waltham, MA, USA) as a competitor and a final concentration of 2 mM DTT. A 1 × reaction buffer (20 mM Tris (pH8), 60 mM KCl, 1 mM EDTA and 12% glycerol (*w*/*v*)) was used for each reaction. EMSAs were run on a 1% Agarose gel containing 0.5 × Tris-Acetate-EDTA (TAE, Foothill Ranch, CA, USA) and a running buffer of 0.5 × TAE for 1 h at 80 V. Signals were visualized using a Thyphoon FLA 9500 instrument (GE Healthcare, Chicago, IL, USA) and analyzed with Quantity One software (Bio-Rad, Hercules, CA, USA). For determination of K_D_ values, the intensity of the “free DNA” was measured using QuantityOne. Data were analyzed by the nonlinear last squares estimate of R fitting data with the Hill equation.

### 2.6. DNase I Protection Assay

PCR-amplified double stranded DNA covering the region 1,440,468–1,440,711 of the *Prochlorococcus* MED4 genome were used as templates. Either the sense or antisense strand was Cy5 labeled using primers # 99–102 with one of both primers being Cy5 labeled in each PCR reaction. Each DNase I footprint reaction contained 2.5 μg of the competitor Lightshift™ Poly dI-dC (Thermo Scientific™, Waltham, MA, USA) in a 1 × reaction buffer comprising 20 mM Tris (pH 8), 60 mM KCl, 1 mM EDTA and 12% glycerol (*w*/*v*), 1 mM DTT and 2 mM MgCl_2_. Two different concentrations (1.3 µM and 2.6 µM) of pMarR together with 375 ng of Cy5 labeled DNA were subjected to 4 units of Turbo DNase I (Thermo Scientific™, Waltham, MA, USA) digestion. Samples were incubated for 4 min at 30 °C. A control reaction omitting pMarR was included. Reactions were terminated by the addition of 0.5 M EDTA and purified using the Macherey-Nagel™ Nucleospin™ Gel and PCR Clean-Up Kit. The sequencing ladders were generated with the same primers as used for the DNase I footprint (# 99–102) using the USB^®^ Thermo Sequenase Cycle Sequencing Kit (Affymetrix) and performed after the manufacturer’s protocol. All samples including the sequencing ladder were air-dried for ~30 min using a speedvac concentrator, subsequently dissolved in 5 μL dionized formamide and incubated for 5 min at 95 °C. The DNase I footprint products and sequencing reactions were separated on 13.3 M urea-8% polyacrylamide (PAA) gels using a 40% solution of acrylamide:bis-acrylamide (19:1). The Rhinohide™ Polyacrylamide Gel strengthener (Thermo Scientific™, Waltham, MA, USA) was added to the 13.3M urea-8% PAA gel with 26.6% of the respective volume of acrylamide:bis-acrylamide (19:1). Signals were visualized using a Typhoon FLA 9500 instrument (GE Healthcare) and analyzed with Quantity One software version 4.6.6 (Bio-Rad).

### 2.7. Protein-Protein Affinity Chromatography and Analysis by Mass Spectrometry

For protein fishing, 2 × 3 L cultures of *Prochlorococcus* MED4 were grown to cell densities of 1–3 × 10^8^ cells/mL. Cell cultures were harvested at 15,000 *g*, for 20 min at 20 °C. Each pellet of a 3-L culture was resuspended in 12.5 mL phosphate buffered saline (PBS, pH 7.4) supplemented with 0.5 mM CaCl_2_ and EDTA-free protease inhibitor (cOmplete™ Protease Inhibitor Cocktail, Roche, Basel, Switzerland). Cells were lysed using the One Shot constant cell disruption system (Constant Systems Limited, Daventry, UK) at 1.8 kbar. To separate cell debris from protein, cells were centrifuged at 13,900 *g* for 30 min at 4 °C and filtered (0.45 µm) into a new falcon tube. The bait proteins were loaded on HisTrap HP columns (Cytiva, Marlborough, MA, USA) and equilibrated with PBS supplemented with 0.5 mM CaCl_2_ on column. Subsequently, the *Prochlorococcus* MED4 cell lysate was incubated with the bait protein on column and protein complexes were eluted with a gradient of washing buffer (5 mM Imidazole, 50 mM NaH_2_PO_4_, 300 mM NaCl, 5 mM NaN_3_) and elution buffer (400 mM Imidazole, 50 mM NaH_2_PO_4_, 140 mM NaCl). Elution fractions were collected in 2-mL Eppendorf^®^ tubes and visualized on a 12% PAA (40% acrylamide: bisacrylamide (37.5:1)) gel by staining with Coomassie blue.

Protein samples from the fractions were precipitated with chloroform/methanol according to Wessel and Flügge [24]. Precipitated proteins were digested in solution using LysC and Trypsin and desalted according to Hammel et al. [25]. Peptides were analyzed on a nanoLC-MS/MS system (eksigent 425 HPLC coupled to a TripleTOF 6600, AB Sciex) as described [26]. Identification and quantitation of proteins from MS data was carried out using MaxQuant Software package v1.6.0.1 [27]. The protein library used for peptide spectrum matching was generated from the NCBI reference sequence (NC_005072.1) and supplemented with sequences for *pmarR* WT and mutant versions as well as a set of 245 common contaminants included in the MaxQuant package. Oxidation of methionine and acetylation of the N-terminus were considered as peptide modifications. Maximal missed cleavages were set to 3, peptide length to 6 amino acids, and the maximal peptide mass to 5600 Da. Thresholds for peptide spectrum matching and protein identification were set by a false discovery rate (FDR) of 0.01. Protein group intensities were normalized to MarR and ranked for their difference in WT vs. mutant MarR G106A. The proteome raw data acquired by MS were deposited to the ProteomeXchange Consortium (proteomecentral.proteomexchange.org (accessed on 20 July 2022)) via the PRIDE partner repository [28] with the dataset identifier PXD035458.

### 2.8. Yeast Two-Hybrid Assay

For yeast two-hybrid assays, vectors containing the GAL4 activation domain (AD) and the GAL4 DNA-binding domain (BD) and strain *Saccharomyces cerevisiae* (*S. cerevisiae*) YH109 (Takara) were used. The transformation of yeast cells was performed as described by the manufacturer using the Frozen-EZ Yeast Transformation Kit II™ (Zymo Research). For selection, yeast cells were plated on complete supplement mixture (CSM) drop out medium (MP Biochemicals) lacking the amino acids tryptophan (trp) and leucine (leu) (CSM -trp -leu) and grown at 30 °C for three to four days. For interaction studies, three individual co-transformants were spotted in serial dilutions on new plates containing CSM -trp -leu or CSM -trp -leu -his supplemented with 5 mM 3-amino1,2,4-triazole (3-AT).

## 3. Results

### 3.1. The Cyanophage Gene Product of PSSP7_011 Is Involved in the Regulation of Host RNase E Gene Expression

**Figure 1:**
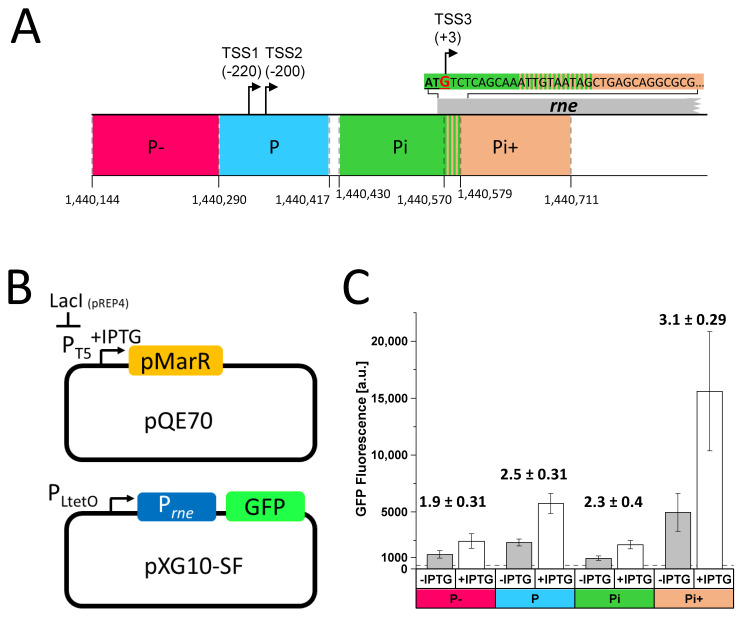
Influence of pMarR transcriptional regulator PSSP7_011 on MED4 *rne* promoter activity. (**A**) Schematic drawing of the *rne* promoter region. The relative locations of TSSs (black arrows and numbers in parentheses) with respect to the start codon (underlined, bold letters) are given. The transcription initiator base G (marked in red) of TSS3 is within the first ATG codon of the full-length CDS. P and Pi correspond to segments associated with TSS1/TSS2 and TSS3, giving rise to the full-length or a truncated form of the *rne* mRNA, respectively. P− is located upstream of the putative *rne* promoter P. The Pi+ segment starts 10 nt upstream of the alternative TSS3 reaching into the downstream region of the CDS. The start and end positions of the tested promoter segments are marked by dashed grey lines and the corresponding coordinates within the MED4 genome are displayed. (**B**) The GFP reporter assay consists of two plasmids: pXG10-SF containing the constitutive P_LtetO_ promoter that is located upstream of the tested *rne* promoter segment, which is fused to a 5′ UTR conferring high expression and the super folder *gfp* gene. pQE70 harbors the gene encoding the transcriptional regulator pMarR under the transcriptional control of the IPTG inducible promoter P_T5_. To reduce the basal expression of *pmarR* prior to induction, *E. coli* Top10 cells also carry the pREP4 plasmid that expresses the LacI repressor for the tight regulation of *pmarR* expression. (**C**) Measurement of GFP fluorescence of *E. coli* cells expressing *sfgfp* under the control of a hybrid promoter that consists of the *E. coli* P_LtetO_ promoter fused to the respective *Prochlorococcus* MED4 P−, P, Pi, or Pi+ promoter segments. GFP expression was carried out under conditions inducing (+IPTG) or non-inducing (-IPTG) expression of *pmarR*. Average fluorescence values were calculated for 50,000 cells per clone from 6 individual clones for each strain. Fold induction relative to −IPTG and respective standard errors were calculated.

A common mechanism to redirect the host transcription machinery to phage transcription is the interaction of a phage-encoded transcription factor with the host RNAP. The only gene in the P-SSP7 genome that encodes a protein with a known transcription factor domain is *pmarR* (PSSP7_011). Therefore, we considered the *pmarR* gene product a promising candidate for controlling *Prochlorococcus* MED4 RNase E gene expression during phage infection. As there is no genetic system for *Prochlorococcus*, we performed heterologous green fluorescent protein (GFP) reporter assays in *E. coli* to test our hypothesis. We analyzed the impact of pMarR on the RNase E promoter region, which contains two tightly spaced TSSs, 220 nt (TSS1) and 200 nt (TSS2) upstream of the start codon, which we refer to as promoter P (genome position 1,440,290–1,440,417), and an alternative TSS3 starting at G (+3) of the canonical ATG start codon with the associated promoter region, which we call Pi (genome position 1,440,430–1,440,579) (Figure 1A). We also analyzed the region upstream of P, which we refer to as P- (genome position 1,440,144–1,440,290) and the region downstream of Pi, which we refer to as Pi+ (genome position 1,440,570–1,440,711), starting 10 nt downstream of TSS3 (Figure 1A). We expressed the pMarR protein from the pQE70 plasmid in the presence of the pXG10-SF plasmid carrying one of the four *rne* promoter segments upstream of the super folder *gfp* gene, which was coupled to a 5′ UTR conferring high expression (Figure 1B). To reduce the basal expression of *pmarR* before induction with IPTG, cotransformation of pQE70 and pXG10-SF was performed with *E. coli* Top10 cells carrying the pREP4 plasmid that expresses the LacI repressor for the tight regulation pQE vectors. Since none of the four *Prochlorococcus* MED4 *rne* promoter segments were recognized by *E. coli* RNAP (Figure S1A), we re-inserted the original promoter of the pXG10-SF expression system, P_LtetO_ upstream of the *rne* promoter regions (Figure 1B) and with this modification we observed GFP fluorescence signals above the threshold for all tested promoter regions in a flow cytometry-based assay (Figure 1C). Interestingly, GFP fluorescence of all four P_LtetO_-*rne* hybrid promoters was elevated in the presence of pMarR (Figure 1C), whereas no differences were observed when we carried out the GFP reporter assay with the empty pQE70 plasmid (Figure S1B). However, we also observed a larger size of *E. coli* cells expressing pMarR when compared to cells that do not express pMarR (Figure S2). Surprisingly, the increase in GFP fluorescence was highest for the promoter containing the Pi+ region (Figure 1C), pointing towards an unusual mode of action of pMarR on a region downstream of the alternative TSS.

### 3.2. DNA-Binding Affinity of pMarR Is Stimulated by Calcium Ions

**Figure 2:**
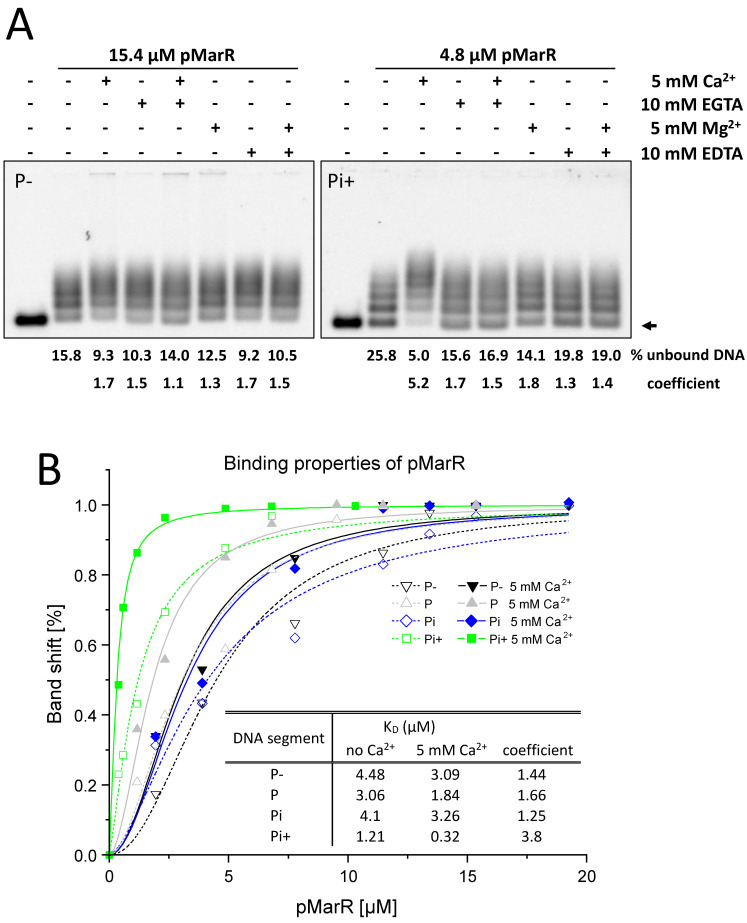
DNA-binding properties of purified recombinant pMarR protein. (**A**) pMarR DNA binding was tested by EMSA using Cy5-labeled *rne* P− and Pi+ DNA segments. Binding reactions contained 15.4 µM (P−) or 4.8 µM (Pi+) recombinant pMarR protein in the presence of 12.5 nM labelled promoter fragment and 1 µg poly (dI-dC) as a non-specific competitor. The influence of divalent cations was tested by the addition of various combinations of Ca^2+^, Mg^2+^, EGTA, and EDTA. The arrow indicates the position of unbound DNA. Shift assays without the addition of divalent cations and chelators were used for the determination of the coefficient that is calculated from amounts of unbound versus bound DNA. (**B**) Binding curves of quantified EMSAs of pMarR protein assaying P−, P, Pi, and Pi+ DNA segments in the absence or presence of Ca^2+^ (Figure S6). Hyperbolic regression lines were generated by fitting with the Hill equation. The inserted table lists K_D_ values calculated based on binding curves. The coefficient represents the factor by which the addition of Ca^2+^ increased the binding affinity.

To investigate the DNA-binding properties of pMarR in more detail, we purified the recombinant pMarR protein by immobilized metal ion chromatography (Figure S3) and performed electrophoretic mobility shift assays (EMSA) with Pi+ and, as a negative control, with P−. Both promoter regions P− and Pi+ were incubated with 15.4 µM or 4.8 µM pMarR protein, respectively, and a fixed amount of Cy5-labeled PCR product. The addition of divalent cations (Ca^2+^ or Mg^2+^) or chelating agents (EGTA or EDTA) did not alter the shift properties in general, indicating that DNA-binding of pMarR is not strictly dependent on divalent cations (Figure 2A). While divalent cations and chelating agents did not the affect binding properties of pMarR to promoter region P−, Ca^2+^ ions largely stimulated the binding of pMarR to Pi+ (Figure 2A). Next, we determined dissociation constants (K_D_) for all four promoter segments in the presence or absence of Ca^2+^ ions (Figure 2B) based on EMSAs that were conducted with a fixed amount of Cy5-labeled PCR product and increasing amounts of purified pMarR protein in the µM range (Figure S4). The lowest K_D_ values, corresponding to the highest affinity, were obtained for the Pi+ DNA fragment with an almost 4-fold lower K_D_ value of 320 nM in the presence of Ca^2+^ ions compared to EMSA conditions without Ca^2+^ (see the table inset in Figure 2B). We conclude that pMarR binds to DNA fragments of different compositions but with different affinities and that these affinities were enhanced in the presence of Ca^2+^ ions, especially for presumably core binding targets.

Next, we examined putative pMarR binding sites within the *rne* promoter by in vitro DNA footprint assays. DNA fragments that encompass the Pi+ and part of the Pi promoter region were generated by PCR with one of both primers labeled with Cy5 for the visualization of the coding or non-coding strand. Labeled DNA fragments were incubated with recombinant pMarR protein in the presence or absence of Ca^2+^ and digested by DNase I (Figure S5). For both strands we observed multiple footprints that cover central parts of the Pi+ region (Figure S5). This observation is consistent with the unusual, ladder-like shift behavior we observed in the EMSA experiments.

### 3.3. Assessment of the Functional Role of Conserved Amino Acids in pMarR

**Figure 3:**
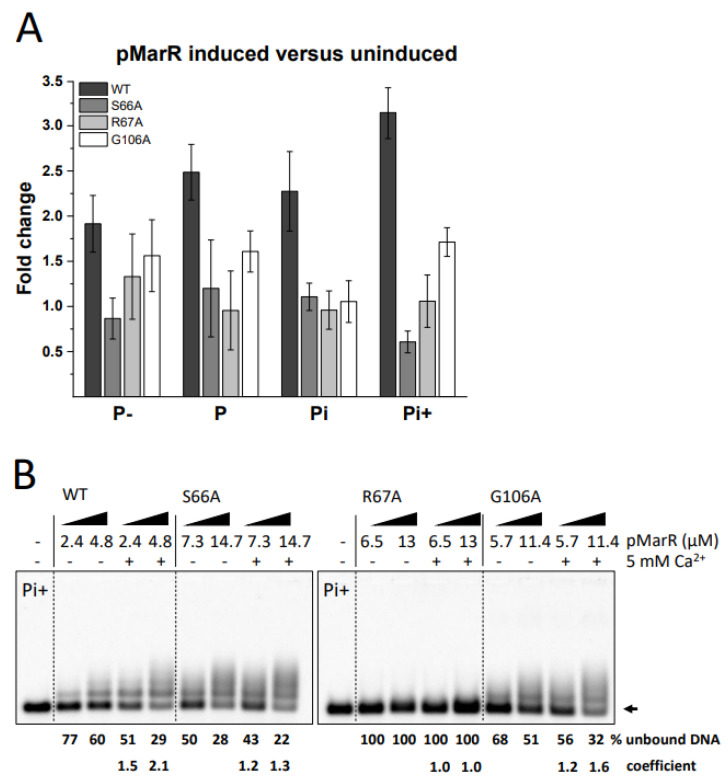
Influence of single amino acid mutations in pMarR on MED4 *rne* promoter activity. (**A**) GFP fluorescence of *E. coli* cells harbouring the pXG10-SF plasmid, whose *gfp* expression is controlled through the analyzed promoter segment and the pQE70 expressing WT or mutants of pMarR. Fold changes were calculated from the average fluorescence values of 50,000 cells per clone from 6 individual clones for each strain, comparing cells that express pMarR (induced with 1 mM IPTG) or do not (uninduced, without IPTG). (**B**) DNA binding of pMarR WT and single amino acid mutants was tested by EMSA using Cy5-labeled *rne* Pi+ DNA segment. Binding reactions contained two concentrations of recombinant pMarR protein in the presence of 12.5 nM labelled promoter fragment, 1 µg poly (dI-dC) as a non-specific competitor, and 0 or 5 mM Ca^2+^. The arrow indicates the position of unbound DNA. Shift assays without Ca^2+^ ions were used for the calculation of the coefficient.

To gain further insight into the interaction between pMarR and the *rne* promoter we used the GFP reporter assay and elucidated the function of several highly conserved residues with putative reactive groups such as carboxy-, guanidine-, or amine groups. Based on a sequence alignment with 77 MarR homologs, we point-mutated 15 amino acid residues to alanine (Figure S6A) and tested the influence of these mutations on the expression of *gfp* from the Pi+ promoter. Among the 15 investigated mutants, three (S66A, R67A, and G106A) showed a considerably lower average fold change between induced and uninduced cells compared to WT pMarR (Figure S6B), suggesting that these amino acids are important for protein activity. The mutants D91A and P92A showed a slightly higher average fold change than WT pMarR (Figure S6B), hence, these substitutions might have locked the protein into an active conformation. Subsequently, the influence of the pMarR S66A, R67A, and G106A mutants on all four *rne* promoter regions was investigated by GFP reporter assays. Compared to WT pMarR, we observed a general decrease in average fold changes for all mutants with all *rne* promoters (Figure 3A). The DNA-binding properties of the mutants were further characterized by EMSA, demonstrating that the purified pMarR R67A protein was not able to shift the Pi+ DNA segment, whereas purified mutant proteins S66A and G106A retained DNA-binding ability (Figure 3B and Figure S3). The addition of Ca^2+^ did not enhance the DNA binding properties of pMarR S66A, whereas a slightly positive effect of Ca^2+^ ions on the retardation properties of pMarR G106A was observed (Figure 3B).

**Figure 4:**
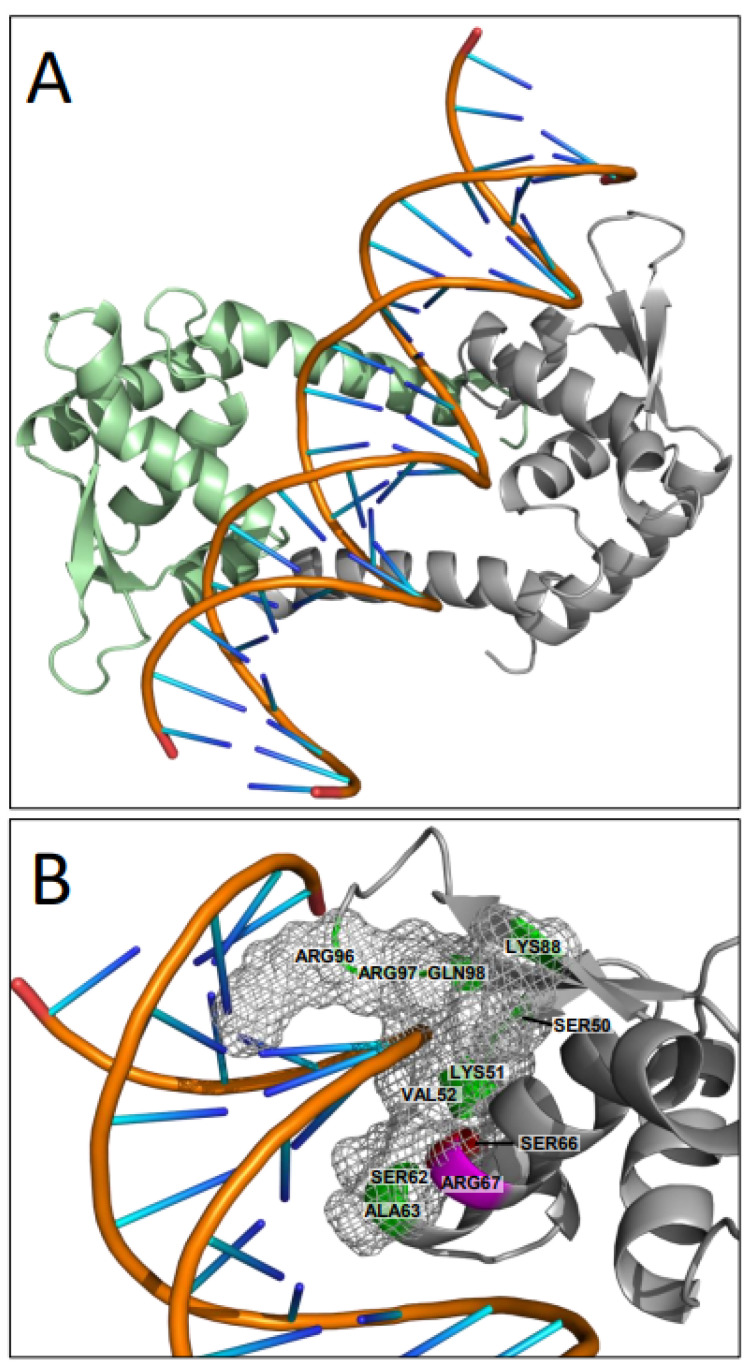
Homology structure model of pMarR-DNA complex. I-TASSER [30] structural und functional prediction of pMarR is based on a tertiary alignment with *Salmonella enterica* virulence regulator SlyA ([31], PDB ID: rq5F). (**A**) Schematic representation of dimeric pMarR (monomers are colored in green and grey) and from the co-crystallized 22-bp DNA duplex containing the SlyA 12-bp high-affinity binding site TTAGCAAGCTAA. (**B**) Putative protein-DNA interaction region. Amino acid residues, visualized as surface mesh, were predicted to interact with DNA (S50, K51, V52, S62, A63, S66, K88, R96, R97, and Q98) and embrace the DNA in a clamp-like structure. In the ribbon representation, predicted DNA-binding residues are colored in green, S66 in red, and R67 in magenta—the latter is not predicted to be involved in the DNA interaction. Structures were drawn with Pymol [32].

To better understand the biological function of residues S66 and R67, we predicted three-dimensional protein structures of dimeric WT pMarR, and the two mutants S66A and R67A (Figure S7) using AlphaFold [29]. As we did not observe any differences in the global protein structures between pMarR WT and the two mutants, we performed a more refined structure-based functional analysis (Figure 4A) using the I-TASSER structure and function predictor [30]. The best ligand prediction for pMarR was nucleic acid with the highest C-score [0, 1] of 0.51 calculated for the structure template of the *Salmonella enterica* MarR family master virulence regulator SlyA [31]. A total of 10 pMarR amino acid residues, including S66, were predicted to be involved in DNA binding, which seem to surround the DNA in a clamp-like structure (Figure 4B). Even though R67 was not predicted by I-TASSER to be involved in DNA interaction, the corresponding arginine residue at position 65 in the SlyA homolog of *Salmonella enterica* plays a central role in DNA binding through the direct recognition of a guanine base [31].

### 3.4. pMarR Interacts with the Alpha Subunit of the Host RNAP

In order to identify potential interacting partners of pMarR in *Prochlorococcus* MED4, we purified recombinant pMarR protein as a bait and serving as a control pMarR mutant G106A. Respective bait proteins were immobilized on nickel-charged affinity resin and incubated with whole-cell lysates from exponentially growing *Prochlorococcus* cells. After washing, bound proteins were eluted using a gradient from 5 mM to 400 mM imidazole and 25 elution fractions were collected and analyzed by SDS-PAGE (Figure S8). The majority of the two bait proteins are eluted in fractions 15 to 25 with the best overlap in abundance in fractions 20 and 21 (Figure S8). Although the majority of the bait protein eluted in later fractions, the imidazole gradient for elution of the bait simultaneously changes the salt concentration of the solution, which can interfere with protein-protein binding. For this reason, fractions 14 and 15 were analyzed to ensure that all potential binding partners were captured. Subsequently, we analyzed the captured proteins of elution fractions 14, 15, 20, and 21 for both pull-downs by mass spectrometry. MS data of the four elution fractions of each pull-down experiment were summarized and for better comparison the pMarR G106 pull-down dataset was normalized based on pMarR WT and pMarR G106 bait protein intensity values. According to intensity values, the respective bait proteins were highly enriched (Table 1 and Table S2). Amongst the enriched proteins that co-eluted with the His-tagged pMarR protein were the four core subunits of the *Prochlorococcus* MED RNAP (*rpoA*, *rpoB*, *rpoC1,* and *rpoC2* encoding subunits α, β, γ, and β’) (Table 1 and Table S2). Notably, we did not detect a sigma factor (Table S2), which possibly may have been lost during the enrichment procedure or sample preparation for mass spectrometry.

We further analyzed the interaction of pMarR with RNAP proteins (PMM1483, PMM1484, PMM1485, PMM1535) and other proteins enriched in the pull-down (PMM0599, PMM1107 (*pdxJ*), PMM1508 (*tufA*)) (Table 1) or proteins of general interest such as the P-SSP7 RNAP (PSSP7_0013), with PMM0486 serving as the negative control, by yeast two-hybrid assays (Figure S9). Because WT pMarR expression seemed toxic to yeast cells, the assays were performed with the DNA-binding deficient mutant R67A (referred to as “WT”) or double mutants R67A_S66A and R67A_G106A, respectively. During restrictive condition in the presence of 5 mM 3-AT, pMarR interacts with itself, to a lesser extent with the S66A mutant, and with the α subunit of the RNAP (Table 2 and Figure S9). The pMarR S66A mutant seems slightly impaired in its capability for protein interactions. The interaction to the WT protein and the α subunit of the RNAP was preserved, while the ability to form homodimers (or multimers) was lost (Table 2 and Figure S9). Notably, pMarR G106A lost the ability to establish any of the interactions that were observed with the WT pMarR or the S66A mutant (Table 2 and Figure S9). For all other tested proteins, including the phage RNAP, we did not detect any interaction with pMarR (Table 2 and Figure S9).

## 4. Discussion

The cyanophage P-SSP7 pMarR transcription factor is involved in the transcriptional regulation of *Prochlorococcus* MED4 host RNase E by promoting a switch from native promoter usage towards an internal promoter. The shorter transcript from the internal promoter is devoid of the autoregulatory cleavage site in the 5′ UTR and produces a shorter RNase E isoform that is catalytically active [17]. We show here that the pMarR transcriptional regulator binds at an unusual location downstream of the TSS. Although transcription factor binding in the path of transcription is rather associated with repressive regulation, heterologous GFP reporter assays showed that pMarR is able to increase protein expression. Transcriptional activation of a binding site downstream of the TSS has been reported for the non-canonical transcription factor GcrA in *Caulobacter crescentus*, where transcription is activated through GcrA binding to RNAP-σ^70^ prior to promoter binding [33]. The RNAP–GcrA complex then binds and activates target promoters harboring a methylated GcrA binding site either upstream or downstream of the TSS [33]. In another study, direct interaction between a transcription factor and the RNAP in combination with a binding site downstream of the TSS has been shown for the AraC family virulence regulator Rns of *E. coli* [34]. Rns has three binding sites in its own promoter P*rns*, one upstream of the TSS, centered at −227, and two downstream of the TSS centered at +43 and +82, and both downstream binding sites are important for guiding the RNAP to the correct position to form an open complex at P*rns* [34]. The synergistic action of the upstream and one of the downstream binding sites was shown to be required for activating transcription from the P*rns* promoter [34] and in combination with crystal structure data it was suggested that Rns might bind to looped DNA [35]. At this point, we do not know if the binding of pMarR involves looped DNA and further studies are necessary to gain deeper insights in the molecular mechanism of the mode of action of pMarR.

Our pull-down and yeast-two hybrid results suggest that pMarR interacts with the α subunit of the RNAP. Interaction between transcription factors and the α subunit of the RNAP have been described for a multitude of transcriptional activators such as CRP, PhoB, and MerR in *E. coli* [36]. However, the binding of pMarR downstream of the TSS should cause steric hindrance as the α subunit is located at the distal site with respect to the polymerization direction of the RNAP. Further studies are needed to elucidate the underlying mechanism of interaction between pMarR and the α subunit of the RNAP.

The ladder-like migration pattern observed in EMSA experiments suggests either a stepwise multimerization of the protein or, more likely, multiple binding sites of pMarR, which is once again unusual in comparison to canonical transcriptional regulators and was observed here for both high and low affinity targets. Exploratory DNA footprint analysis indeed revealed multiple binding sites for pMarR with possibly a seed region in the Pi+ region that may explain the greater affinity compared to the other tested promoter regions. However, we cannot exclude that the multimerization might be an experimental artefact that might not occur in in vivo situations where the ratio of pMarR to target DNA might be lower.

We were also able to identify important amino acids for core functions of pMarR. The ultraconserved (conserved in 75 of 77 compared proteins) arginine at position 67 is essential for DNA binding. The importance of a conserved arginine in the DNA binding domain of a *Prochlorococcus* MED4 transcription factor was observed before for PMM1637 [22], and arginine also has been demonstrated to be crucial for DNA binding of the *Salmonella enterica* virulence factor SlyA [31]. The heterologous GFP reporter system also showed that glycine at position 106 was essential for the functionality of the protein. Compared to WT pMarR, affinity to DNA seemed reduced in the G106A mutant but unlike the S66A mutant, the Ca^2+^ sensitivity seemed preserved. Therefore, the loss-of-function in the G106A mutation must be via a different mechanism. The yeast two-hybrid assays and the protein-protein interaction pulldown experiments revealed that G106 is likely essential for dimerization and that the dimer form of pMarR is required for interaction with the RNAP. However, further studies are required to gain a better understanding of the protein-protein interaction of pMarR and RNAP. In the direct vicinity of R67 is serine at position 66, which increased the affinity to DNA in a Ca^2+^-dependent manner. A requirement of Ca^2+^ ions during phage infection has been previously reported. For instance, Ca^2+^ is essential for the entry of the marine bacteriophage PM2 into the *Pseudoalteromonas* sp. ER72M2 host cell as genomic DNA penetration through the cytoplasmic membrane depends on the presence of Ca^2+^ ions [37]. The prerequisite of Ca^2+^ ions not only for DNA injection but also for phage propagation has been reported for the Mycobacteriophage I3 infecting *Mycobacterium smegmatis* SN2 [38]. Whether Ca^2+^ is simply a cofactor or if Ca^2+^ signaling might be important during phage infection, is a promising starting point for future work.

## Figures and Tables

**Table 1:** Top list of proteins identified by LC-MS analysis, sorted by greatest difference in abundance between pMarR WT and pMarR G106A protein pull-down experiments. Intensity values of pMarR G106A pull-down data were normalized based on pMarR WT and pMarR G106 bait protein intensity values.

Protein IDs	Intensity WT	Intensity G106A (Normalized)	Difference WT-G106A
rpoC2 (PMM1483)	587,230	894	586,336
rpoB (PMM1485)	477,070	3316	473,754
rps19 (PMM1554)	904,660	462,906	441,754
eno (PMM0208)	332,800	2638	330,162
PMM0855	339,410	15,255	324,155
dapL (PMM1500)	326,460	34,569	291,891
pdxJ (PMM1107)	237,270	0	237,270
tufA (PMM1508)	387,360	244,715	142,645
rps17 (PMM1549)	160,920	36,521	124,399
rpoA (PMM1535)	97,779	0	97,779
rbcL (PMM0549)	97,015	393	96,622
rpoC1 (PMM1484)	93,962	0	93,962
rpl27 (PMM1345)	119,740	30,858	88,882
PMM0599	88,057	0	88,057
bait protein	2,051,700	2,051,700	0

**Table 2:** Overview of the tested interactions between pMarR and potential interaction partners by yeast two-hybrid assays. Experiments were conducted in biological triplicates (Figure S9) and numbers correspond to the number of replicates where cell growth was observed. Individual co-transformants were spotted in serial dilutions in 10-fold increments on plates containing CSM +His or CSM -His supplemented with 5 mM 3-amino1,2,4-triazole (3-AT). Interactions observed in three, two, or one replicate(s) are marked in dark green, light green, and yellow, respectively.

	No 3-AT, +His			+3-AT, No His		
Dilution Factor	1	10^−1^	10^−2^	1	10^−1^	10^−2^
	WT_R67			WT_R67		
WT_R67	3	3	3	3	2	1
S66_R67	3	3	3	3	2	0
G106_R67	3	3	2	0	0	0
*rpoC2* (PMM1483)	3	3	2	0	0	0
*rpoC1* (PMM1484)	3	3	2	0	0	0
*rpoB* (PMM1485)	3	3	2	0	0	0
*rpoA* (PMM1535)	3	3	3	3	2	0
PMM0486	3	3	3	0	0	0
PMM0599	3	3	2	0	0	0
*pdxJ* (PMM1107)	3	3	3	0	0	0
*tufA* (PMM1508)	3	3	2	0	0	0
*gp1*, RNAP (PSSP7_013)	3	3	2	0	0	0
	S66_R67			S66_R67		
WT_R67	3	3	2	2	1	0
*rpoA* (PMM1535)	3	3	1	3	1	0
	G106_R67			G106_R67		
WT_R67	3	3	3	0	0	0
*rpoA* (PMM1535)	3	3	2	0	0	0

## Data Availability

The datasets generated and/or analyzed during the current study are available in the ProteomeXchange Consortium via the PRIDE partner repository with the dataset identifier PXD035458. Reviewers can access data using this link: Available online: https://www.ebi.ac.uk/pride/archive/login (accessed on 20 July 2022); Username: reviewer_pxd035458@ebi.ac.uk; Password: WUCEcfsC.

## Supplementary Materials

The following supporting information can be downloaded at: https://www.mdpi.com/article/10.3390/microorganisms10112245/s1. Reference [39] are cited in the supplementary materials.

## Abbreviations

Endoribonuclease E	RNase E
RNA polymerase	RNAP
transcriptional start site	TSS
