# Supplementary material for: A Cyanophage MarR-Type Transcription Factor Regulates Host RNase E Expression during Infection
Source: Microorganisms. 2022 Nov 13;10(11):2245. doi: 10.3390/microorganisms10112245 (PMC9692554; doi:10.3390/microorganisms10112245)
Supplement: Supplementary file 1 [file microorganisms-10-02245-s001.zip › Supplementary material_new.pdf]

Supplementary material

Table S1. List of oligonucleotides used in this study.

Table S2. List of identified proteins by MS analysis.

Figure S1. GFP reporter assays. A) With pXG10-SF constructs devoid of the native P_{LtetO} promoter. GFP expression is driven by *Prochlorococcus* MED4 P-, P, Pi and Pi+ promoter segments. pQE70 harbours the transcriptional regulator pMarR under the transcriptional control of P_{T5}. B) Measurement of GFP fluorescence of *E. coli* cells expressing the empty pQE70 plasmid, and *sfgfp* under the control of a hybrid promoter that consists of the *E. coli* P_{LtetO} promoter fused to the respective *Prochlorococcus* MED4 P-, P, Pi or Pi+ promoter segments. GFP expression was measured in cells expressing (induction with 1 mM IPTG) pMarR or in cells under non-inducing conditions (without IPTG). Average fluorescence values were calculated for 50,000 cells per clone from six individual clones for each strain. The variation of fluorescence of the individual clones is given by error bars.

Figure S2. Assessment of *E. coli* cell size by flow cytometry. Forward angle light scatter (FSC-A) signals of *E. coli* cells harboring the pXG10-SF plasmid with promoter element P-, P, Pi or Pi+ and pQE70 with pMarR under induced (+IPTG, black lines) or uninduced (-IPTG, blue lines) conditions. For each *rne* promoter construct one representative clone is shown. The FSC signal intensity is proportional to cell size, primarily due to light diffraction around the cell.

Figure S3. Purification of recombinant pMarR WT and mutant proteins. SDS-PAGE analysis of recombinantly expressed pMarR WT, S66A, R67A and G106A. Recombinant proteins were purified from *E. coli* cell lysate by immobilized metal ion chromatography. To visually evaluate concentrations of purified proteins, increasing amounts (black triangles) were loaded on the gel. The gel was stained with Coomassie blue.

Figure S4. pMarR DNA-binding affinities to *rne* promoter regions P-, P, Pi and Pi+. A) pMarR DNA binding was tested by EMSA using Cy5-labeled *rne* P-, P, Pi+ and Pi+ DNA segments without addition of Ca^{2+}. Binding reactions contained increasing amounts of recombinant pMarR protein in the presence of 12.5 nM labelled promoter fragment and 1 µg poly (dI-dC) as a non-specific competitor. The arrows indicate the position of unbound DNA. B) The same EMSA experiments as in A) in the presence of 5 mM Ca^{2+} (+).

Figure S5. DNase I protection by pMarR bound to a 244 nt *rne* promoter segment that encompasses central parts of the Pi+ region. DNA footprints of Cy5-labelled DNA fragments of the A) coding and B) non-coding strand covering the region 92 nt upstream (partial Pi) and 151 nt downstream (Pi+) of the alternative *rne* TSS3 with 0 µM, 1.3 µM or 2.6 µM pMarR and without (-) or in the presence of 5 mM Ca^{2+} (+). Colored parentheses show the positions of pMarR binding sites and the blue rectangle indicates the position of TSS3. C) Corresponding sequence region visualized in the 13.3 M urea-8% PAA gels in A) and B). The black arrow marks the position of TSS3 and the vertical dashed lines the boarders of the Pi and Pi+ fragments. Detected footprints of the coding and non-coding strand are boxed with the same colors as in A) and B).

Figure S6. GFP reporter assay testing the influence of single amino acid mutations in pMarR on the expression of GFP from the Pi+ promoter *in vivo*. A) Sequence logo of 77 MarR family transcriptional regulators. Sequences were aligned using the muscle algorithm and the logo was generated using WebLogo [1]. Numbers within the logo refer to the alignment position. Mutated amino acids and positions within pMarR are indicated by red arrows. B) Fold changes of induced versus non-induced *E. coli* cells expressing pMarR WT or single amino acid mutants in the presence of the pXG10-SF plasmid containing the Pi+ promoter and, as a control, pMarR WT in the presence of the pXG10-SF plasmid containing the P- promoter. Average fluorescence values were calculated for 50,000 cells per clone from six individual clones for each strain. Fold induction relative to -IPTG and respective standard errors were calculated.

Figure S7. Overlay of homology models of dimeric pMarR WT (yellow ocher) S66A (medium purple) and R67A (cyan). Structures were created using AlphaFold [2].

Figure S8. SDS-PAGE analysis of protein pulldown experiments of pMarR WT and pMarR G106A. Recombinant proteins were purified from *E. coli* cell lysate by immobilized metal ion chromatography. Elution profiles of the collected elution fractions (numbers on top of gel) are shown. M – marker, L – lysate loaded on column, FT – flow through. The positions of the recombinant proteins in PAA gels are marked by red arrows.

Figure S9. Interaction studies between pMarR and potential interaction partners by yeast two-hybrid assays. Individual co-transformants of *S. cerevisiae* YH109 cells were spotted in serial dilutions in 10-fold increments on plates containing CSM +His (control plates) or CSM -His supplemented with 5 mM 3-AT (selective plates). For each combination three individual clones were grown at 30°C for three to four days. The red ellipse marks a contamination originating from the interaction tested above.

References

1. Crooks, G.E.; Hon, G.; Chandonia, J.-M.; Brenner, S.E. WebLogo: A Sequence Logo Generator. *Genome Res.* **2004**, *14*, 1188–1190. https://doi.org/10.1101/gr.849004.
2. Jumper, J.; Evans, R.; Pritzel, A.; Green, T.; Figurnov, M.; Ronneberger, O.; Tunyasuvunakool, K.; Bates, R.; Žídek, A.; Potapenko, A.; et al. Highly Accurate Protein Structure Prediction with AlphaFold. *Nature* **2021**, *596*, 583–589. https://doi.org/10.1038/s41586-021-03819-2.
