# Supplementary figures and images for: A Cyanophage MarR-Type Transcription Factor Regulates Host RNase E Expression during Infection
Source: Microorganisms. 2022 Nov 13;10(11):2245. doi: 10.3390/microorganisms10112245 (PMC9692554; doi:10.3390/microorganisms10112245)

A

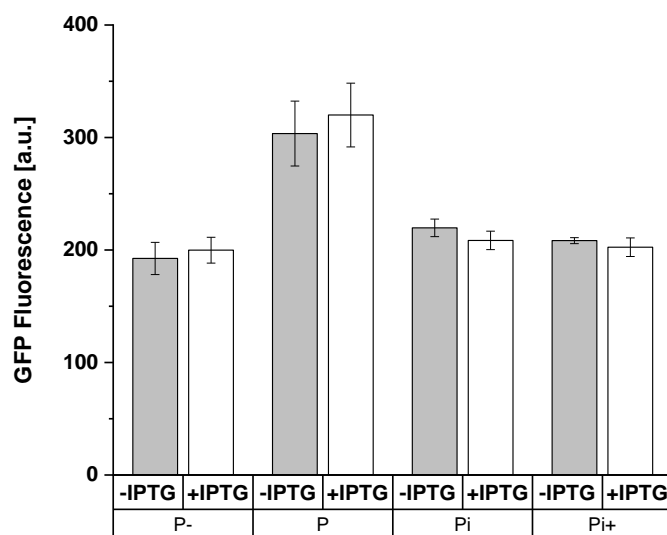

B

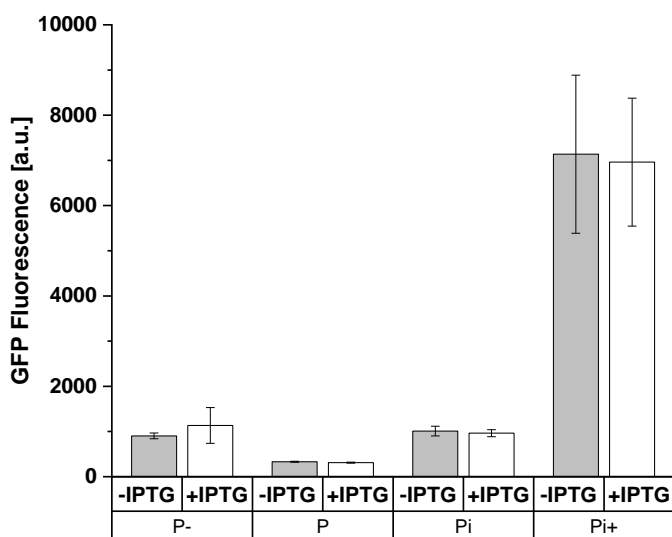

Figure S1

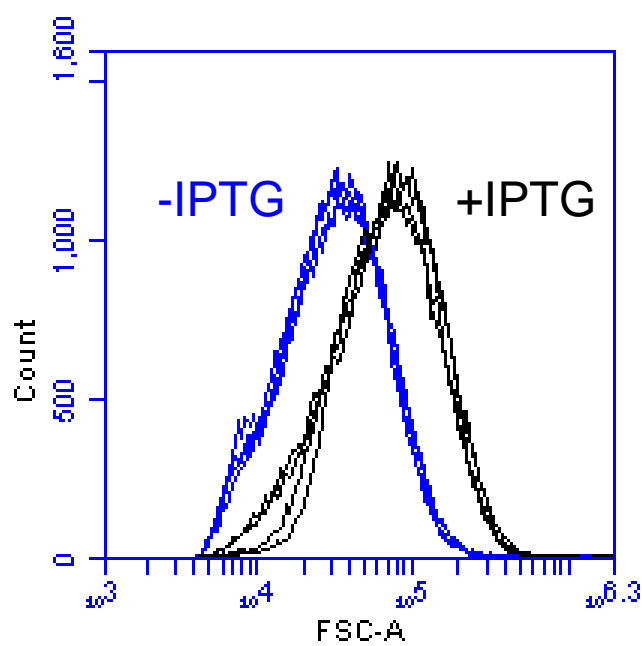

Figure S2

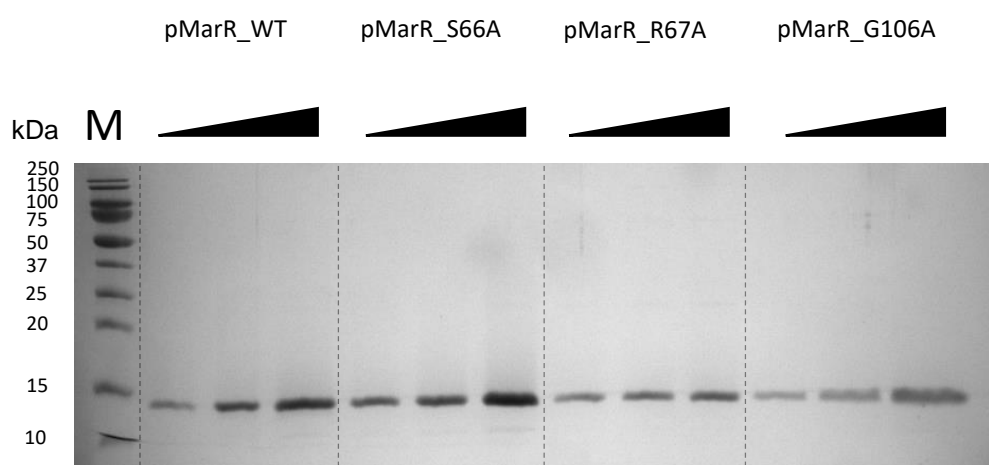

Figure S3

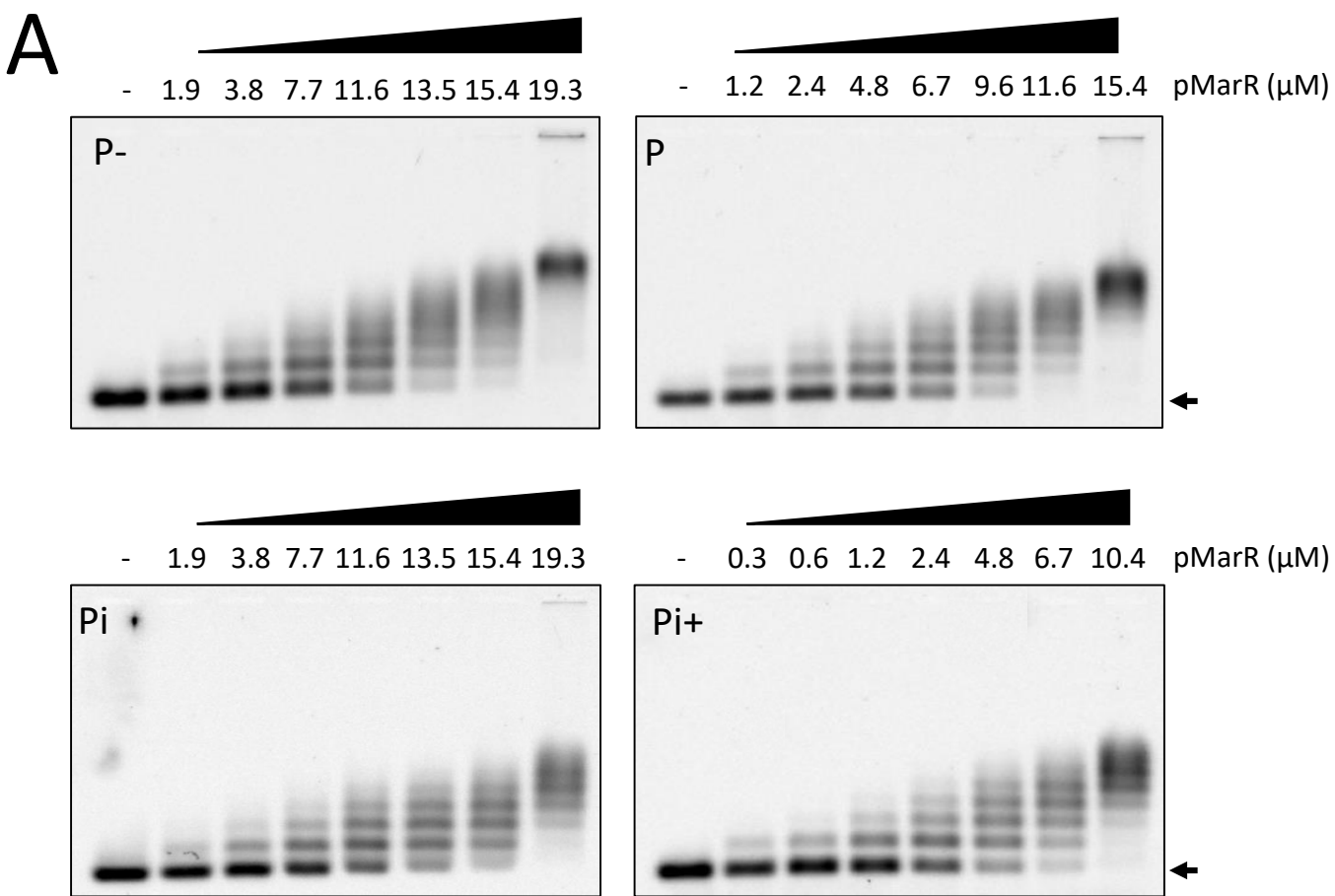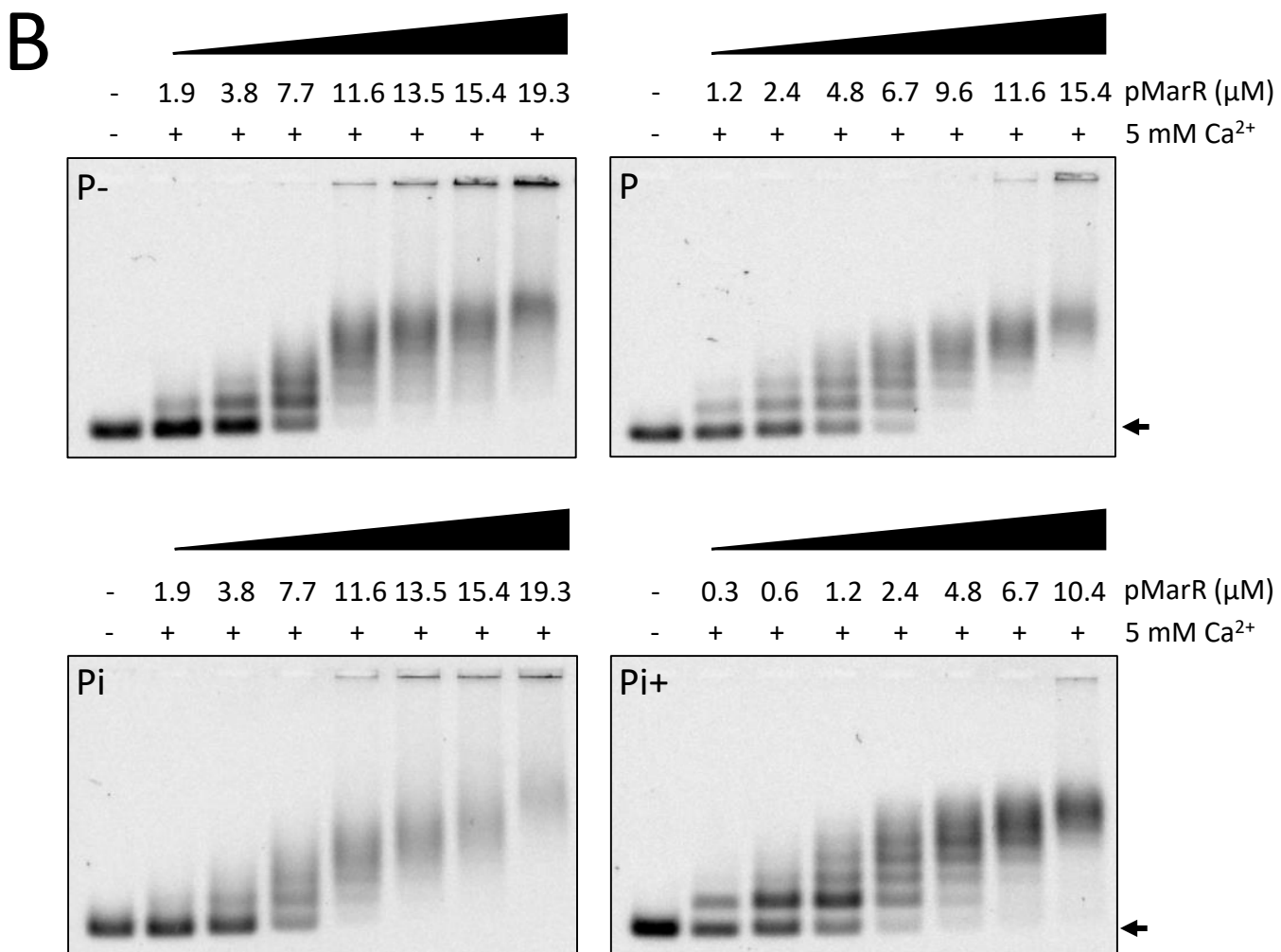

Figure S4

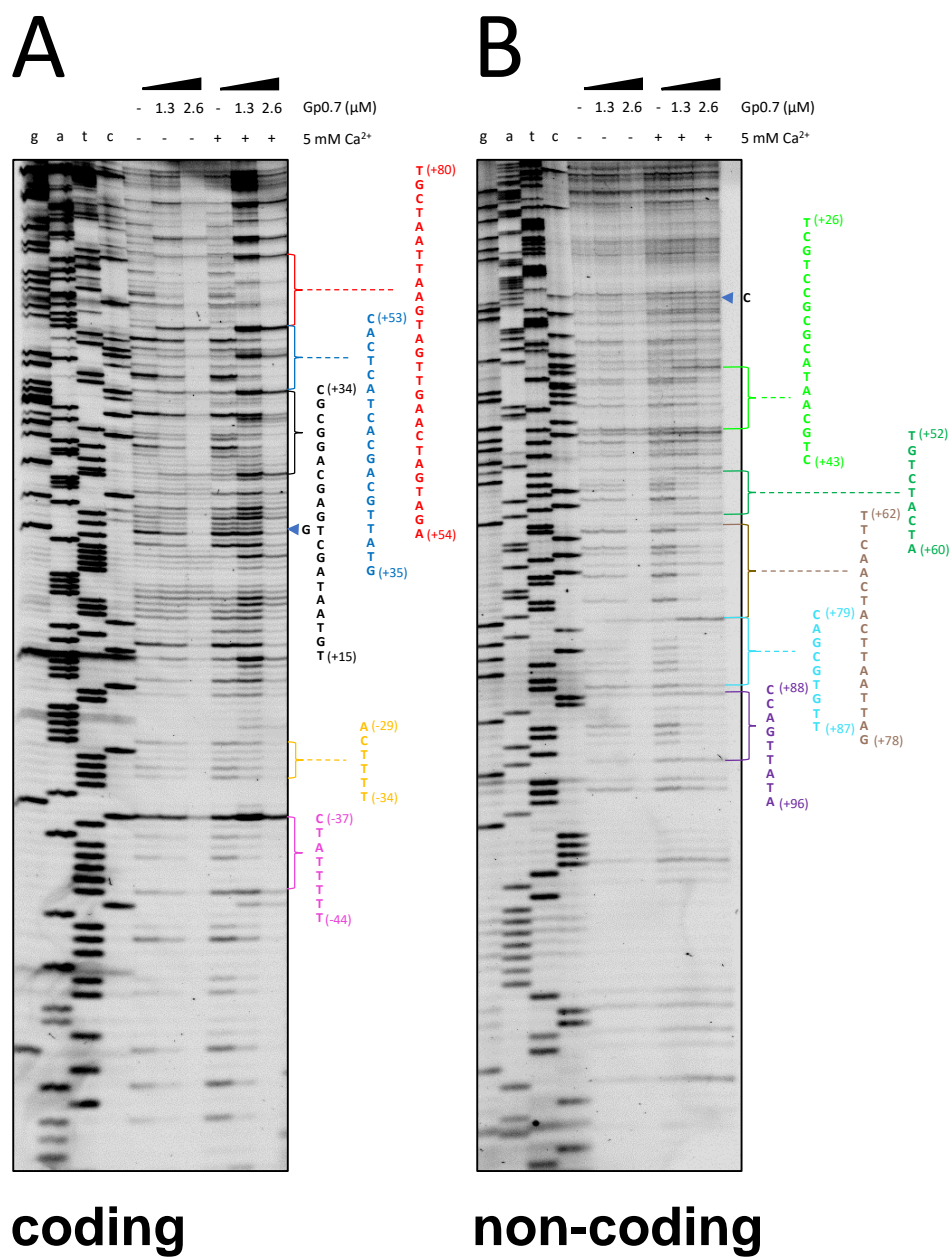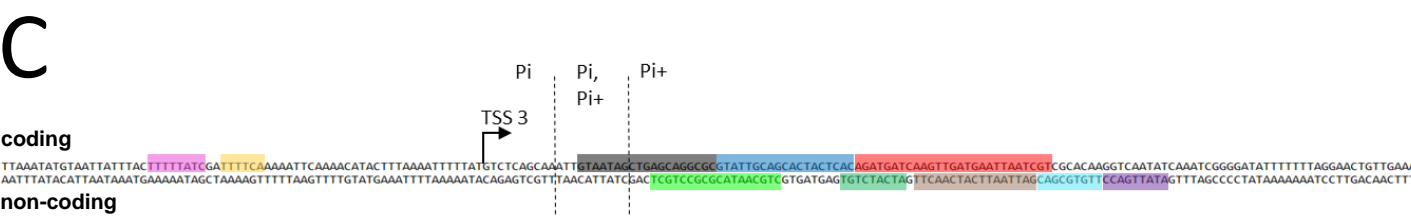

Figure S5

# A

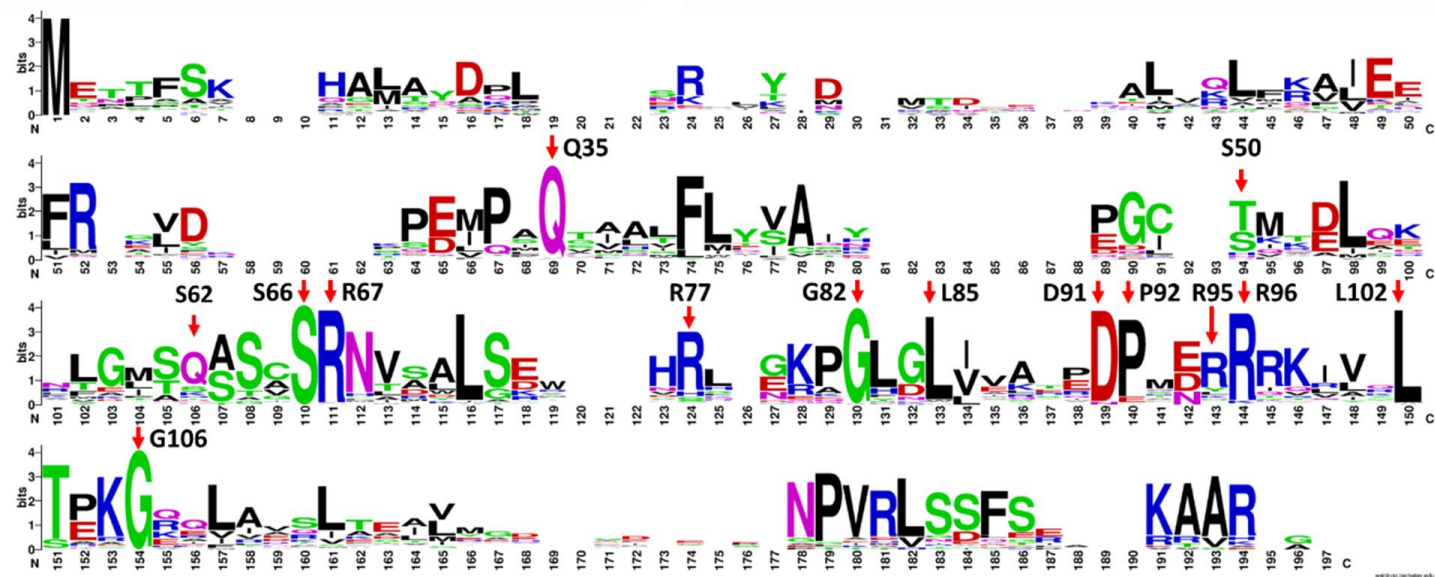

# B

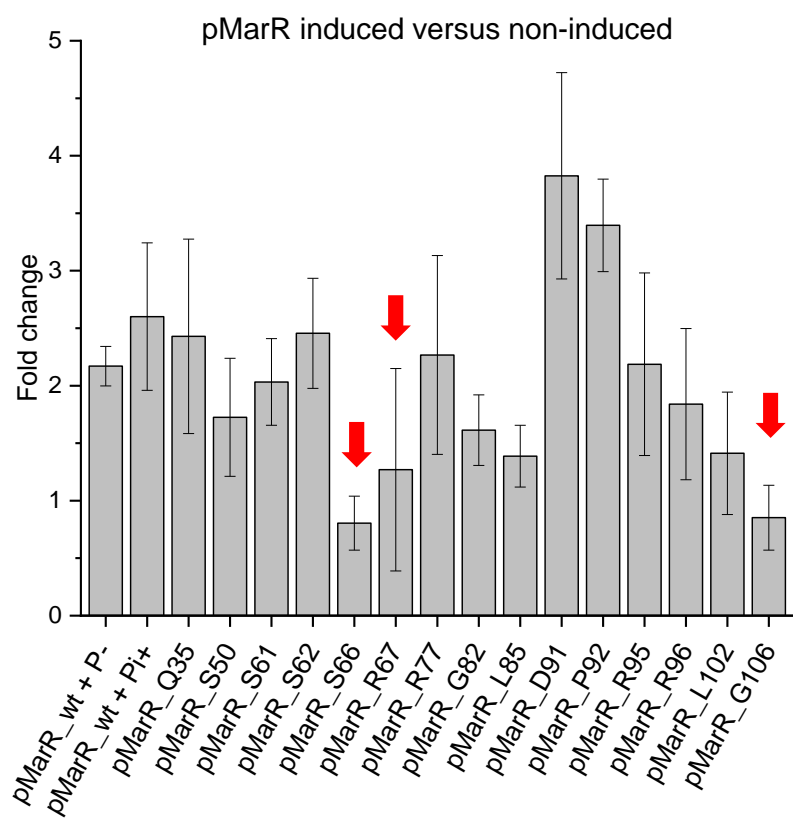

Figure S6

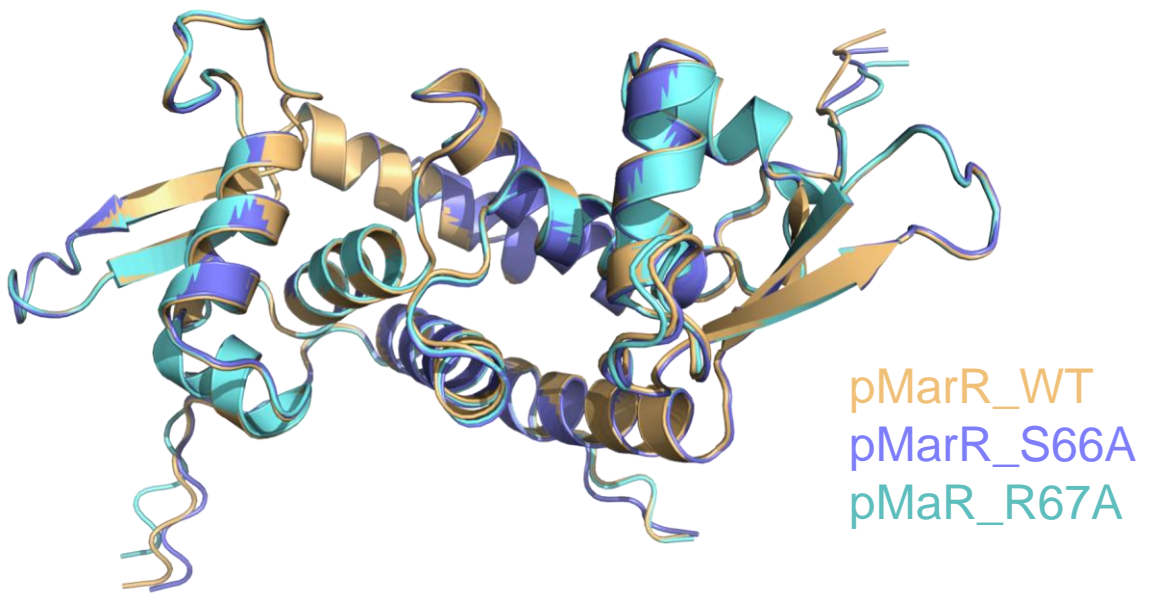

Figure S7

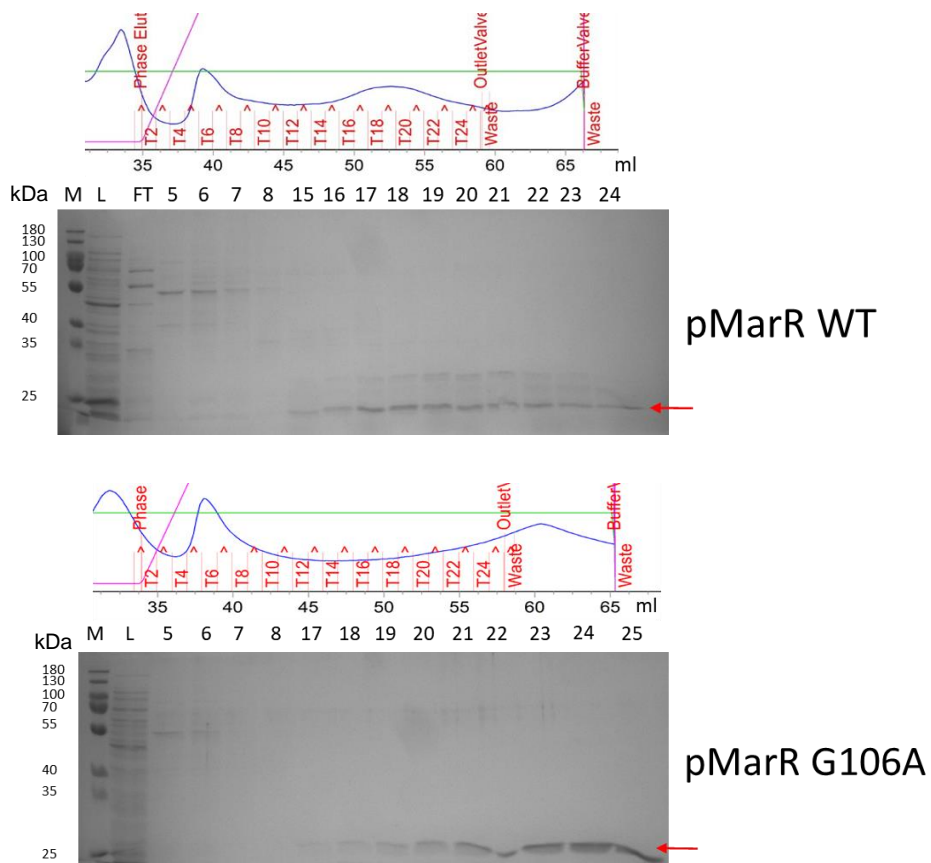

Figure S8

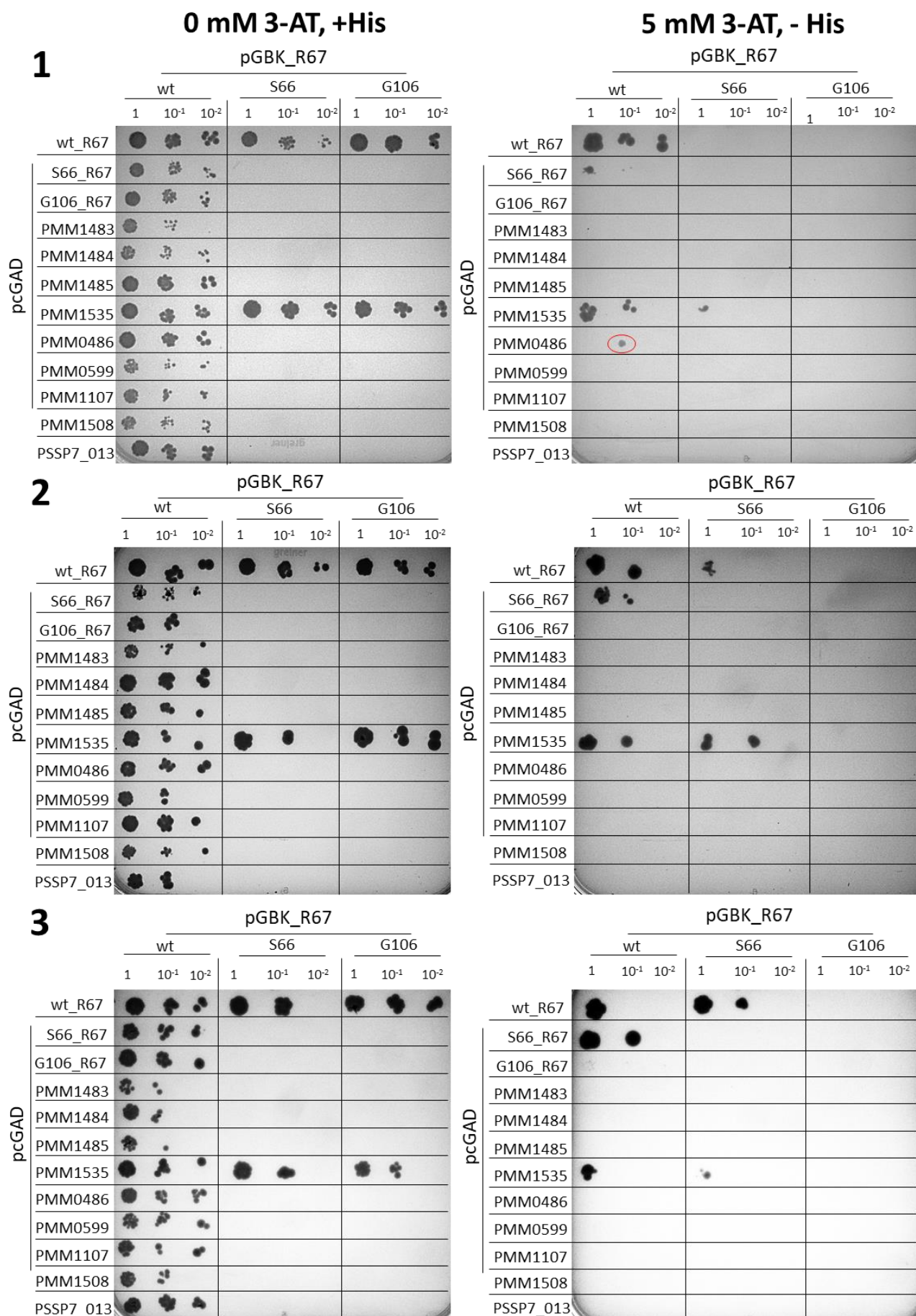

Figure S9

Supplement: Supplementary file 1 [file microorganisms-10-02245-s001.zip › Suppl_Figures.pdf]
